# Effectiveness of elexacaftor-tezacaftor-ivacaftor in people with cystic fibrosis carrying non-F508del variants in Brazil

**DOI:** 10.36416/3086-3910/e20250384

**Published:** 2026-03-05

**Authors:** Mariane Gonçalves Martynychen Canan, Caroline Souza Sokoloski, Paulo de Tarso Roth Dalcin, Raphael Freitas Jaber de Oliveira, Bruna Ziegler, Samia Zahi Rached, Débora Carla Chong e Silva, Luiz Vicente Ribeiro Ferreira da Silva, Leonardo Araújo Pinto, Rodrigo Abensur Athanazio

**Affiliations:** 1. Serviço de Pneumologia, Complexo Hospital de Clínicas, Universidade Federal do Paraná, Curitiba (PR) Brasil.; 2. Serviço de Pneumologia, Hospital de Clínicas de Porto Alegre, Universidade Federal do Rio Grande do Sul, Porto Alegre (RS) Brasil.; 3. Policlínica Universitária Piquet Carneiro, Universidade Estadual do Rio de Janeiro, Rio de Janeiro (RJ) Brasil.; 4. Serviço de Fisioterapia, Hospital de Clínicas de Porto Alegre, Universidade Federal do Rio Grande do Sul, Porto Alegre (RS) Brasil.; 5. Divisão de Pneumologia, Instituto do Coração, Faculdade de Medicina, Universidade de São Paulo, São Paulo (SP) Brasil.; 6. Divisão de Pneumologia Pediátrica, Complexo Hospital de Clínicas, Universidade Federal do Paraná, Curitiba (PR) Brasil.; 7. Unidade de Pneumologia Pediátrica, Instituto da Criança e do Adolescente, Faculdade de Medicina, Universidade de São Paulo, São Paulo (SP) Brasil.; 8. Programa de Pós-Graduação em Medicina - Pediatria, Escola de Medicina, Pontifícia Universidade Católica do Rio Grande do Sul, Porto Alegre (RS) Brasil.

## TO THE EDITOR:

The advent of cystic fibrosis transmembrane conductance regulator (CFTR) modulators established a therapeutic milestone in the treatment of people with cystic fibrosis (PwCF), with significant clinical and functional improvement.^1^ These benefits have been particularly evident since the U.S. Food and Drug Administration (FDA) approved the triple combination of elexacaftor, tezacaftor, and ivacaftor (ETI) for people with CF with at least one F508del mutation, in 2019. In the following years, the FDA expanded ETI indication to 271 additional variants, most of which were supported by in vitro studies using Fischer rat thyroid (FRT) cells. 

Approximately 82% of people with CF worldwide carry at least one F508del mutation.^2^ In Brazil, however, this group accounts for < 70% of the CF population.^3^ This highlights the marked genetic heterogeneity of Brazilian people with CF and underscores a substantial proportion of individuals not covered by the initial approval. 

Subsequent real-world studies confirmed that many variants approved on the basis of in vitro evidence showed sustained clinical response in vivo, reinforcing the relevance of FRT cell models.^2^ ^,^ ^4^ Moreover, in vivo response to ETI has also been demonstrated in PwCF with non-FDA approved variants, despite negative in vitro results.^2^ ^,^ ^4^ ^,^ ^5^ In this context, this case series aims to describe the clinical impact of ETI use in Brazilian PwCF carrying non-F508del variants, including mutations without prior evidence of in vitro responsiveness. 

We conducted a retrospective case series analysis describing the clinical evolution of people with CF with non-F508del mutations treated with ETI. Seven CF referral centers participated in the study, with data obtained during routine clinical visits conducted between 2022 and 2025. Evaluations were performed at baseline and 12-16 weeks after initiation of treatment with ETI. Clinical and demographic variables were collected, including *CFTR* genotypes, percent predicted FEV_1_ (FEV_1_%), BMI, sweat chloride concentration, respiratory symptoms, and pulmonary exacerbations. Adverse events were monitored throughout the study period. The study project was approved by the local research ethics committee (Protocol no. 15748619.8.2052.5327), and written informed consent was obtained from all participants or their legal guardians. 

A total of 34 PwCF, aged 10 to 49 years, were evaluated (Table 1). In most individuals, non-F508del mutations were either homozygous or heterozygous with an intrinsically nonresponsive class I mutation, allowing analysis of ETI in 15 variants (G85E, N1303K, A234D, P205S, H1375P, R668C, G551D, 2789+5G>A, S589N, 3272-26G>A, R334W, A561E, R1066C, R1066H, and 875+1G>A). Notably, 8 of the 10 most common non-F508del, non-class I variants in Brazil were analyzed at least once in this paper.^3^

**Table 1:** Demographic and clinical characteristics of 34 patients with cystic fibrosis before and after 12-16 weeks of treatment with ETI.

Patient	Age (years)	Sex	Genotype	CFTR modulator at ETI initiation	FEV_1_ (% predicted)		Sweat chloride (mmol/L)		BMI (kg/m^2^)		Number of exacerbations (oral)*		Number of exacerbations (i.v.)*		Long-term oxygen therapy		Noninvasive ventilation		Adverse events
					Before	After	Before	After	Before	After	Before	After	Before	After	Before	After	Before	After	
1	20	female	R1066C / I507del	None	17	34	91	48	18.5	21.9	3	1	1	0	yes	no	no	no	no
2	27	female	N1303K / G542X	None	28	42	NA	94	18.2	19.5	2	0	1	1	no	no	no	no	acne
3	22	male	G85E / 541del4	None	45	64	NA	NA	17.3	18.3	0	1	1	0	no	no	no	no	mild increase in alkaline phosphatase
4	35	male	G85E / R1162X	None	26	33	100	68	18.4	21.3	0	1	1	0	no	no	no	no	anxiety; moderate increase in CPK
5	46	female	G85E / R334W	None	37	42	97	43	24.0	25.2	2	1	1	0	no	no	no	no	no
6	26	female	H1375P / G542X	None	39	52	86	9	16.6	18.4	2	0	0	0	no	no	no	no	abdominal pain; moderate increase in CPK
7	23	female	G85E / 1812-1G>A	None	37	42	84	57	17.6	20.7	0	0	0	0	no	no	no	no	no
8	13	male	A234D / G542X	None	105	127	92	21	19.5	23.2	2	0	0	0	no	no	no	no	mild increase in alkaline phosphatase
9	43	female	D1152H / A234D	None	69	70	59	15	21.2	22.4	1	0	0	0	no	no	no	no	no
10	10	male	N1303K / R1162X	None	37	38	90	74	13.6	14.8	1	0	0	0	no	no	no	no	no
11	39	female	A561E / A561E	None	37	44	124	87	19.8	19.8	0	0	1	0	no	no	no	no	no
12	10	female	A561E / S466X	None	78	86	92	62	13.1	14.9	2	0	0	0	no	no	no	no	mild liver enzyme increase; rash
13	10	female	A561E / S466X	None	68	78	94	65	13.7	15.7	2	0	0	0	no	no	no	no	mild liver enzyme increase; rash
14	29	female	R334W / 3120+1G>A	None	25	32	116	96	16.4	17.3	1	0	1	0	yes	no	no	no	rash
15	33	female	P205S / CFTRdele14b	None	46	56	85	51	19.9	20.7	0	0	0	0	no	no	no	no	no
16	47	female	875+1G>A / 1717-1G>A	None	37	45	82	49	17.6	18.6	1	0	0	0	no	no	no	no	mild increase in bilirubin
17	19	female	G85E / Y1092X	None	32	48	98	70	24.8	25.62	1	0	1	0	no	no	no	no	no
18	19	male	G85E / G85E	None	72	95	87	66	23.0	25.0	2	0	1	0	no	no	no	no	mild liver enzyme increase; moderate increase in CPK
19	24	female	G85E / Y1092X	None	37	52	98	65	19.6	23.0	2	0	0	0	yes	no	no	no	mild liver enzyme increase; moderate increase in CPK; acne; rash
20	24	female	S589N / G542X	None	21	23	97	75	18.6	20.5	1	0	3	1	yes	yes	yes	no	mild liver enzyme increase; rash
21	26	male	R334W / G542X	None	52	56	86	65	23.5	24.2	1	0	0	0	no	no	no	no	mild liver enzyme increase
22	27	male	G85E / R334W	None	34	41	92	70	23.2	24.8	1	0	2	0	no	no	no	no	blood pressure elevation
23	32	female	3272-26G>A / R1162X	None	36	42	78	54	28.0	28.8	2	1	1	0	no	no	no	no	no
24	21	female	R668C / 2143delT	None	50	54	74	NA	16.5	20.9	2	1	1	0	no	no	no	no	rash
25	45	female	R334W / G542X	None	22	31	NA	NA	21.4	22.7	2	2	4	0	yes	yes	yes	no	no
26	28	male	G551D / G542X	Ivacaftor	23	20	NA	NA	16.5	17.9	1	0	1	0	yes	yes	no	no	no
27	49	female	P205S / W1282X	None	23	30	61	20	24.5	25.3	1	1	1	0	yes	no	no	no	no
28	46	male	G85E / R1162X	None	20	34	NA	NA	23.7	26.2	1	0	2	0	no	no	no	no	mild liver enzyme increase
29	49	female	2789+5G>A / R1162X	Ivacaftor	25	29	89	NA	21.0	21.0	2	1	2	0	yes	yes	no	no	no
30	26	female	R1066H / 405+1G>T	None	52	62	NA	NA	16.3	18.1	1	1	1	0	no	no	no	no	no
31	27	male	R1162X / N1303K	None	50	76	93	92	21.9	22.3	0	0	0	0	no	no	no	no	no
32	8	female	G85E / G542X	None	69	88	103	58	14.8	17.2	1	0	2	0	yes	no	no	no	no
33	10	female	R1066C / R1066C	None	90	101	81	24	16.9	18.4	1	0	1	1	no	no	no	no	no
34	14	female	N1303K / 1078delT	None	79	94	79	NA	18.8	20.9	3	1	0	0	no	no	no	no	no

CFTR: cystic fibrosis transmembrane conductance regulator; CPK: creatine phosphokinase; ETI: elexacaftor-tezacaftor-ivacaftor; and NA: not available. *Four-month evaluation before and after ETI use.

All patients demonstrated improvement in at least one outcome. A reduction of respiratory symptoms was observed in all individuals,; 88% experienced a BMI gain ≥ 0.5 kg/m^2^; 79.4% achieved an increase of ≥ 5% in FEV_1_%; and 94% showed a reduction ≥ 20 mmol/L in sweat chloride concentration. No significant adverse events were reported. Observed benefits and safety profiles were comparable to those in F508del studies.^1^

Regarding pulmonary function, 7 PwCF exhibited only minimal FEV_1_% gain. Such heterogeneity in response to ETI was reported in one study with F508del patients, of whom approximately 10% had a variation < 2.5% in FEV_1_% despite improvement in other clinical endpoints.^6^ This may reflect the limited impact of ETI on lung function when severe structural pulmonary damage is present. Notably, 4 of our patients with limited lung function response had advanced lung disease, including 2 who had previously been treated with ivacaftor. 

Another relevant aspect is the impact of ETI on sweat chloride levels, whose reduction reflects CFTR restoration. Lower post-ETI values have recently been associated with better clinical outcomes.^7^ In our series, nearly 50% of individuals with available sweat test results achieved values < 60 mmol/L, which is the diagnostic threshold for CF, and 5 reached normal levels. In those with the N1303K variant, sweat chloride reduction was minimal despite clinical improvement. This finding, which was also observed in R334W and 2789+5G>A carriers, may reflect a genotype-dependent variation in CFTR expression or regulation across different cell types.^2^ Though all R334W individuals in our series showed clinical improvement, post-ETI sweat chloride levels remained elevated. This finding reinforces that, while sweat chloride concentration is an important biomarker, it may not consistently reflect treatment response across all *CFTR* variants. 

Although quality of life was not formally assessed, respiratory symptoms and pulmonary exacerbations are known to reduce overall well-being in CF patients, with the latter also contributing to disease progression and mortality.^8^ Despite the short evaluation period in the present study, respiratory symptoms and pulmonary exacerbations declined substantially. After ETI, outpatient-treated pulmonary exacerbations decreased by 72.1% and hospitalizations decreased by 89.7%, exceeding the improvements reported in F508del heterozygotes.^1^ In addition, long-term oxygen therapy use decreased by 55.6%, and the two patients receiving noninvasive ventilation discontinued its use. Comparable results were reported in F508del patients with advanced lung disease treated with ETI, supporting ETI, supporting its efficacy in PwCF with non-F508del mutations.^9^

Twenty-five individuals in the present series had at least one FDA-approved variant, with approval largely based on studies using FRT cells. Clinical improvement was observed in 1-2 patients with A234D, P205S, H1375P, R668C, G551D, 2789+5G>A, S589N, R1066H, and 3272-26G>A variants. For the G85E and N1303K mutations, which accounted for 14 PwCF in this cohort, response was documented in ≥ 3 patients. These findings are consistent with previously published data, confirming that FRT cell responsiveness correlates strongly with clinical benefit.^2^ One variant, namely, D1152H, was only analyzed in heterozygous state with another responsive mutation, precluding individual interpretation of its response profile. 

In contrast, the responses of R334W, R1066C, and A561E variants to ETI have previously been reported only in real-life studies.^2^ ^,^ ^5^ Interestingly, although these variants are uncommon in international registries, they are among the most common non-F508del mutations in Brazil and were considered nonresponsive on FRT cells assays.^3^ ^,^ ^4^

R334W is the most common non-F508del, non-class I mutation in Brazil.^3^ We observed ETI response in 3 PwCF carrying this variant, with 2 showing an FEV_1_% gain > 5% and all 3 demonstrating a BMI increase ≥ 2 kg/m^2^. These results are consistent with those reported in the expanded French compassionate program for ETI use.^2^ Similarly, R1066C is also frequent in the Brazilian pwCF and has been described as a responsive variant in real-world studies.^2^ ^,^ ^5^ Both patients with this variant in this paper had an increase > 10% in FEV1% and sweat chloride levels below 60 mmol/L after treatment. We also observed 3 PwCF with the A561E mutation, which has previously been described in only a single real-world clinical case.^2^. These 3 patients achieved an FEV1% gain ≥ 7% and a sweat chloride reduction of 29-37 mmol/L after ETI treatment. Collectively, these findings confirm the consistent clinical responsiveness of these variants to ETI, despite negative in vitro results. 

Finally, ETI provided clinical benefit in one CF patient with the 875+1G>A splicing mutation, a rare minimal function variant not previously described individually at the time of writing. Despite the association between this mutation and very limited CFTR production,^10^ the patient showed clinical improvement across all assessed domains. 

Our study reinforces the effectiveness of ETI in CF patients with non-F508del variants, a finding of particular relevance in genetically admixed populations such as the Brazilian one. It also highlights the value of in vivo assessment for rare variants that cannot be evaluated in clinical trials, supporting access to effective treatment for a broader number of people with CF. 

In conclusion, this case series provides real-world evidence supporting the use of ETI in people with CF with non-F508del mutations, demonstrating improvements in lung function, BMI, sweat chloride concentration, and respiratory symptoms at the individual level. Our cohort included 11 FDA-approved variants and four non-FDA-approved variants (R334W, A561E, R1066C, and 875+1G>A), with clinical responses observed after 12-16 weeks of treatment. 
